# Lessons from Ten Years of Validation of Targets Identified by the Accelerating Medicines Partnership in Alzheimer’s Disease (AMP‐AD)

**DOI:** 10.1002/alz.091089

**Published:** 2025-01-03

**Authors:** Michelle E. Ehrlich

**Affiliations:** ^1^ Icahn School of Medicine at Mount Sinai, New York, NY USA

## Abstract

**Background:**

The Accelerating Medicines Partnership in Alzheimer’s Disease (AMP‐AD) is a public‐private partnership linking NIH, the FDA, pharmaceutical companies, and nonprofit organizations in an interactive, collaborative program utilizing transcriptomics, genomics, metagenomics, proteomics, and metabolomics to provide data for computational analysis, that, in turn, enables promising targets to be ranked by a combination of omic scores and druggability. This ranking informs the selection of targets for validation.

**Method:**

Human postmortem samples were obtained from Mount Sinai, ROSMAP (Religious Orders Study and Rush Memory and Aging Project), Mayo Clinic (Florida), and Columbia University. The validation systems were mouse and fly models of AD pathology and human induced pluripotent stem cells (hiPSCs). Target genes were up‐ or down‐ regulated or mutated to determine whether AD‐related phenotypes were altered.

**Result:**

Targets selected for validation by AMP‐AD included genes expressed in neurons (e.g., VGF, REST, ATP6V1A), microglia (e.g., BIN1, TYROBP, TREM2, SYK, MSN, INPP5D), or astrocytes (e.g., PLEXNB1, GJA1). As expected, phenotypes were improved by decreasing levels of proteins that are abnormally elevated in AD or by increasing the levels of proteins that are abnormally decreased in AD. Qualitative and quantitative variability in effects of target manipulation may be attributable to duration or stage of disease at the time of intervention, and/or to the cell type that expresses each target. AD‐related changes were used to predict therapeutic directionality, but up‐ and down‐regulation sometimes had similar effects. Plaque load changes did not reliably predict changes in other aspects of the phenotype. Paradoxically, manipulation of some targets (e.g., ABI3) caused amyloidosis and tauopathy to change in different directions.

**Conclusion:**

Validation experiments in multiple systems support the AMP‐AD approach for selecting druggable targets for the treatment of AD. Future work will focus on validation of additional targets and close collaboration with the Target Enablement to Accelerate Therapy in AD (TREAT‐AD) Consortium to develop pharmacologic agents derived from these discoveries.

